# Integration of Copper Toxicity Mechanisms in *Raphidocelis subcapitata*: Advancing Insights at Environmentally Relevant Concentrations

**DOI:** 10.3390/toxics12120905

**Published:** 2024-12-13

**Authors:** Manuela D. Machado, Eduardo V. Soares

**Affiliations:** 1 Bioengineering Laboratory, ISEP, Polytechnic of Porto, Rua Dr António Bernardino de Almeida, 431, 4249-015 Porto, Portugal; mmmachado@net.sapo.pt; 2 CEB—Centre of Biological Engineering, University of Minho, Campus de Gualtar, 4710-057 Braga, Portugal; 3 LABBELS—Associate Laboratory, 4800-122 Braga, Portugal

**Keywords:** antioxidative defenses, cell membrane integrity, lipid peroxidation, oxidative stress, photosynthetic activity, *Pseudokirchneriella subcapitata* (=*Selenastrum capricornutum*)

## Abstract

This work aimed to characterize the impact of copper (Cu), at environmentally relevant concentrations, using the freshwater microalga *Raphidocelis subcapitata*. Algae were incubated with 33 or 53 µg/L Cu, in OECD medium, and toxic impacts were evaluated over 72 h, using different cellular and biochemical biomarkers. The exposure to 33 µg/L Cu had an algistatic effect: slowing growth and reducing algal population (53%, at 72 h) without compromising the cell membrane. This Cu concentration promoted a transient reduction in chlorophyll *a* (chl*a*) content and typical markers of oxidative stress: increased levels of reactive oxygen species (ROS), augmented catalase (CAT) activity, and lipid peroxidation (malondialdehyde, MDA). Algae exposed to 53 µg/L Cu, suffered a severe effect with a 93% reduction in the number of cells, 50% decrease in chl*a* content, and diminished (17%) maximum photochemical quantum yield of PSII (*F*_v_/*F*_m_). This population also presented increased levels of ROS and MDA, 33 and 20 times higher than the control, respectively, at 72 h, augmented CAT activity, and permeabilized cell membrane (5%, at 72 h). These findings provide valuable insights into Cu toxicity in aquatic ecosystems, highlighting the biochemical and physiological impacts at environmentally relevant concentrations.

## 1. Introduction

Copper (Cu) is an important environmental pollutant, being released into the environment either due to natural sources (e.g., windblown dusts, decaying vegetation, or forest fires) or anthropogenic activities. Sources of Cu in aquatic environments include copper mining leachate, industrial effluents (such as electroplating), Cu-based pesticides, algaecides, marine paints, and domestic sewage treatment plants [1]. Therefore, Cu concentrations in ground waters vary from 0.2 to 98 µg/L [2]. In surface waters, a range from 50 to 610 µg/L has been described [3]. Higher Cu concentrations were observed in surface waters in places near abandoned mines or that receive water drainage from mining operations; in these cases, Cu concentration can reach 69 mg/L [1].

The negative impact of Cu on the aquatic environment is documented, as well as its tendency to accumulate throughout the food chain [4,5], presenting potential hazards to humans [1,6]. Microalgae, as producers (trophic level 1), are a primary target of the toxic action of Cu. In this regard, the action of Cu on growth inhibition and reduction in photosynthetic activity of marine diatom *Phaeodactylum tricornutum* [7], and green microalgae *Chlamydomonas reinhardtii* [8], *Chloromonas augustae* [9], *Dunaliella salina* [10], *Scenedesmus* sp. [11], *Raphidocelis subcapitata* [12,13,14], and *Selenastrum gracile* [15] has been described. Cu triggered the loss of membrane integrity in *P. tricornutum* [16], *Chlorella sorokiniana* [17], *Scenedesmus acuminatus* [17], and *R. subcapitata* [13], and oxidative stress biomarkers in *C. reinhardtii* [8,18,19,20,21], *Chorella vulgaris* [22], *C. augustae* [9], *R. subcapitata* [22,23], and *Scenedesmus* sp. [24]. Cu also induces the inhibition of esterase activity and damage of mitochondrial function in *R. subcapitata* in a dose-dependent manner [13,25,26,27] as well as induces changes in the expression levels of transcripts that encode for enzymes associated with several metabolic pathways (such as photosynthesis and glycolysis) and oxidative stress response in *C. reinhardtii* [28,29] and in the amino acid profile and proteome in *Chlorella* sp. [30].

The present work aims to evaluate the impact of Cu, at sub-lethal and environmentally relevant concentrations, on the physiology of the freshwater unicellular microalga *R. subcapitata* (formerly known as *Selenastrum capricornutum* and *Pseudokirchneriella subcapitata*), a well-known model organism, widely used in toxicity assessment [31] but poorly exploited in the elucidation of the mechanisms of action of toxicants, namely Cu. For this purpose, a kinetic and holistic approach was used, which combined different cellular and biochemical biomarkers of toxicity, such as photosynthetic pigments content, photosynthesis activity, cell membrane integrity, and oxidative stress indicators: level of reactive oxygen species (ROS), membrane lipid peroxidation, and antioxidant enzymes (catalase and superoxide dismutase). As the result of the integration of the knowledge obtained from the different endpoints evaluated, a mechanism of action (toxicity pathway) of Cu, at environmentally relevant concentrations, on the freshwater alga *R. subcapitata* was proposed.

## 2. Material and Methods

### 2.1. Alga and Cultural Conditions

The unicellular alga *Raphidocelis subcapitata* was sourced from the Culture Collection of Algae and Protozoa (CCAP), Dunbeg, UK. The alga was cultivated in the OECD medium [32]. For strain maintenance, algal cells were grown in OECD medium with 2% (*w*/*v*) of agar (Merck, Darmstadt, Germany) and stored at 4 °C. Cultures were obtained by the inoculation of 5 × 10^4^ cells/mL in 400 mL of OECD medium, followed by incubation at 25 °C, with shaking (100 rpm), for 48 h under continuous “cool white” fluorescent light (fluorescent lamps with a color temperature of 4300 K) with an intensity of 54 µmol photons/m^2^/s at the surface of the flasks.

Algae concentration was measured using an automatic cell counter (TC10, Bio-Rad, Hercules, CA, USA).

### 2.2. Exposure of Alga to Copper and Sampling Process

For evaluating the effects of Cu in *R. subcapitata*, algae were exposed to Cu concentrations that fulfill the following criteria: (*i*) low enough to correspond to levels found in the aquatic environment (environmental relevance) and (*ii*) have a major impact on cell growth. After an initial study, the nominal concentrations of 33 and 53 µg/L Cu were selected. Therefore, appropriated volumes of a standard solution (Merck, in water) of 2000 mg/L Cu(NO_3_)_2_ were added to algal cells, in the exponential phase of growth, at 1 × 10^5^ cells/mL, in the OECD medium, at pH 7.5, contained in 1L-glass Erlenmeyer flasks (total volume: 400 mL). The cultures were later incubated under the conditions described above. Control cultures (i.e., without Cu extra addition, beyond that present in the OECD medium) were also prepared.

Free Cu concentrations, present in the culture medium, were calculated using the MINEQL+ software (version 4.5) [33]. Simulation studies were carried out at pH 7.5, wherein total Cu and ligands concentrations were considered as well as the affinity constants between Cu and the different ligands and the respective solubility product constants, obtained from Martell and Smith [34]. Thus, free Cu concentrations were estimated to be 1.4 × 10^−8^ in control cultures (i.e., without Cu extra addition) and 2.3 and 5.1 µg/L in cultures where nominal concentrations of 33 and 53 µg/L Cu were, respectively, added.

After 24, 48, and 72 h of incubation, samples were harvested, and the algal population was measured as described above. Then, in the remaining part of the samples, the cells were centrifuged at 2500× *g* and resuspended in 100 mmol/L phosphate-buffered saline (PBS), at pH 7.0, to determine ROS production and lipid peroxidation. Alternatively, cells were resuspended in OECD medium for the quantification of cell viability, photosynthetic pigments content, and evaluation of photosynthetic activity. To determine the activity of antioxidant enzymes, the cells were resuspended in a lysis buffer whose composition is described in the section “Antioxidant enzymes activity determination”.

### 2.3. Determination of Photosynthetic Pigments

For the quantification of the photosynthetic pigments, 5 mL of algal suspension (3 × 10^6^ cells/mL) was centrifuged at 2500× *g* for 10 min. After the removal of the supernatant, algae cells were resuspended in 5 mL of 90% (*v*/*v*) acetone (VWR Chemicals, Fontenay-sous-Bois, France) and the extraction of pigments occurred at 4 °C, for 20 h. Then, cells were harvested by centrifugation, at 2500× *g*, for 10 min, and the absorbance of the supernatant was measured at 630, 647, 664, and 750 nm for chlorophyll *a* (chl*a)* determination, and 480 nm for carotenoids quantification, as described by Ritchie [35] and Strickland and Parsons [36], respectively. For each time and experiment, pigments concentration was determined in triplicate.

### 2.4. Assessment of Photosynthetic Activity

The photosynthetic activity of photosystem II (PSII) was evaluated using a pulse-amplitude-modulated (PAM) chlorophyll fluorometer (Junior PAM, Walz, Effeltrich, Germany). Cells (3 × 10^6^/mL to ensure a dense cell layer at the bottom of the tube, providing accurate and reliable measurements) were, firstly, dark-adapted for 30 min. Then, the minimum (*F*_0_) and the maximum fluorescence (*F*_m_) yield were measured, thus enabling the calculation of the maximum photochemical quantum yield of PSII (*F*_v_/*F*_m_). To assess the efficiency of light energy utilization, a continuous actinic light (190 µmol photons/m^2^/s) with saturating pulses every 20 s for 5 min was applied to algal cells to obtain minimum and maximum fluorescence (*F*′_0_ and *F*′_m_, respectively). These parameters allowed the calculation of the proportion of light absorbed by chlorophyll associated with PSII that is used in photochemistry (Φ_PSII_), the rate of electron transport through PSII (*ETR*), and the absorbed light energy dissipated as heat from PSII, non-photochemical quenching (*NPQ*) [37]. All parameters were evaluated in each time and experiment, and were calculated using the WinControl software (version 3.2.2).

### 2.5. Quantification of ROS Production

ROS production was monitored with 2′,7′–dichlorodihydrofluorescein diacetate (H_2_DCFDA, Sigma-Aldrich, St. Louis, MO, USA). Algal cells (1 × 10^6^/mL) were incubated, in the dark, with H_2_DCFDA in a final concentration of 10 µmol/L for 90 min, as previously described [23]. In each time and experiment, fluorescence intensity was measured in quintuplicate, as relative fluorescence units (RFUs), in a microplate reader (Victor3, Perkin-Elmer, Cridersville, OH, USA) at a fluorescence excitation wavelength of 485/14 nm and an emission of 535/25 nm. Fluorescence was corrected by subtracting the autofluorescence of cells, PBS buffer, and fluorescent dye.

### 2.6. Antioxidant Enzymes Activity Determination

To gain a deeper understanding of the effect of Cu on the antioxidant defense system of *R. subcapitata*, superoxide dismutase (SOD), and catalase (CAT) enzymes were determined. At times indicated in the figures, cells were harvested by centrifugation (5000× *g*, for 5 min, at 4 °C) and resuspended (at 1–2 × 10^7^/mL) in an iced lysis buffer with the following composition: 100 mmol/L PBS, pH 7.0, with 1 mmol/L ethylenediaminetetraacetic acid (EDTA) and 0.5 mmol/L phenylmethylsulfonyl fluoride (PMSF). Cell suspension (0.5 mL) was placed in a 2 mL microtube containing 0.3 g of glass beads (425–600 µm of diameter) and lysed in a bead-beating grinder (FastPrep-24^TM^ 5G, MP Biomedicals, Irvine, CA, USA), using 10 cycles of 8.5 m/s for 45 s, with 2 min intervals between each cycle for cooling the samples on ice. The lysates were centrifuged (3220× *g*, for 15 min, 4 °C), and the supernatants were used for enzyme determination.

SOD activity was quantified by the xanthine-oxidase-cytochrome *c* method, described by McCord and Fridovic [38]. The reaction mixture contained 1.4 mL of 50 mmol/L PBS, pH 7.8, 0.1 mmol/L EDTA, 0.01 mmol/L cytochrome *c* (cyt *c*) and 0.05 mmol/L xanthine was combined with 0.05 mL of cell extract and 0.05 mL of 0.005 U of xanthine oxidase. As a control, an uninhibited reaction (without cell extract) was used to obtain the highest rate of reduction in cyt *c*. The reaction was initiated by the addition of xanthine-oxidase and the increase in absorbance at 550 nm was followed for 5 min. One unit of SOD activity is defined as the amount of enzyme that gives 50% inhibition of the control rate of cyt *c* reduction. At each time and experiment, SOD activity was determined in triplicate and was expressed as Units (U) per 10^6^ cells.

CAT activity was determined following the procedure described by Aebi [39]. The decline in absorbance at 240 nm is correlated with the breakdown of H_2_O_2_ by catalase. The diminishing of H_2_O_2_ was monitored for 5 min in a 3.00 mL reaction mixture consisting of 1.25 mL of 50 mmol/L PBS, pH 7.0, with 0.1 mmol/L EDTA, 1.00 mL of 30 mmol/L H_2_O_2_ (in 50 mmol/L PBS, pH 7.0), and 0.75 mL of cell extract. One unit of CAT activity is defined as the amount of enzyme which decomposes 1 μmol of H_2_O_2_ per min, under the test conditions. The reaction starts with the addition of H_2_O_2_. At each time and experiment, CAT activity was determined in triplicate and was expressed as Units (U) per 10^6^ cells, calculated using the molar extinction coefficient of 43.6 L/mol/cm.

### 2.7. Quantification of Lipid Peroxidation

Lipid peroxidation in algal cells was quantified using a thiobarbituric acid reactive substances (TBARS) assay [40], as described by Buege and Aust [41]. Briefly, 0.5 mL of algal suspension (1 × 10^8^ cells/mL) was mixed with 1.0 mL of TCA-TBA-HCl reagent [15% (*m*/*v*) of trichloroacetic acid (TCA); 0.375% (*m*/*v*) of thiobarbituric acid (TBA); 0.25 mmol/L HCl]. The mixture was placed in a bath at 95 °C, for 45 min. Then, the mixture was cooled at room temperature and centrifuged (2500× *g*, for 15 min) to remove interferences. The absorbance of the supernatant was read at 532 nm and TBARS were expressed as malondialdehyde (MDA) equivalents. MDA concentration was calculated using the extinction coefficient 156 L/mM/cm [41] and expressed as nmol MDA/10^6^ cells. At each time and experiment, MDA content was evaluated in triplicate.

### 2.8. Evaluation of Cell Membrane Integrity

Cell membrane integrity was evaluated using the fluorescent probe SYTOX green (SG). The probe (SG) penetrates cells with a permeabilized plasma membrane but is excluded from cells with an intact membrane. Algae (at 1 × 10^6^ cells/mL) were stained with 0.5 µmol/L SG (Molecular Probes, Eugene, OR, USA) for 20 min, at 25 °C, in the dark [42]. At least four samples of 100 cells (*n* ≥ 400 cells) were counted in randomly selected microscope fields for each experiment and time. Cells were observed using an epifluorescence microscope equipped with a light-emitting diode (LED) illumination system (pE-400, CoolLED, Andover, UK) and a filter set GFP from Leica.

### 2.9. Reproducibility of the Results and Statistical Analysis

All experiments were repeated, independently, under identical conditions, three to six times. The data are presented as the mean ± standard deviation (SD). Statistical analyses were performed using Kruskal–Wallis test followed by Dunn’s post hoc test multiple comparison method or unpaired *t* test, as described in the legend of the figures; *p* values < 0.05 were considered statistically significant.

## 3. Results

### 3.1. Effect of Cu on Alga Proliferation Capacity

**Figure 1:**
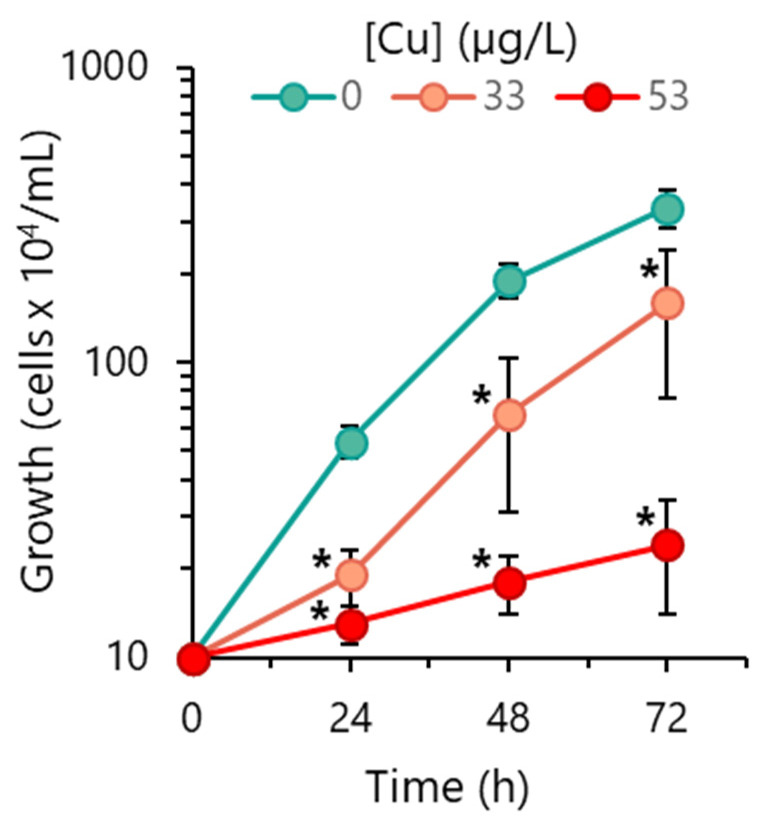
Effect of Cu on *R. subcapitata* proliferation capacity. Evolution of algal population on OECD medium in the absence (control) or the presence of Cu. Data are presented as mean values ± SD (error bars). At each time, the statistical difference between control and Cu-treated cells was tested using unpaired Student’s *t*-test; the means with (*) are significantly different from the control (*p* < 0.05, *n* = 6).

*R. subcapitata* was exposed to low and environmentally relevant concentrations of Cu: 33 and 53 µg/L. For comparative purposes, cells were grown in OECD medium without an extra addition of Cu (control). In control cultures, algae grew exponentially during 48 h, with a specific growth rate (µ) of 0.061/h, which corresponded to a doubling time (generation time), t*_d_*, of 11.4 h. A growth slowdown occurred between 48 and 72 h (Figure 1).

Cu deeply impacted the alga growth, even at a minute concentration. The exposure to 33 and 53 µg/L Cu resulted in a decrease in the number of cells after 72 h of 53 and 93%, respectively, compared to the control (Figure 1).

The evaluation of algal concentration over time allowed for the verification that 33 µg/L Cu severely reduced algal growth during the first 24 h (Figure 1). After this time, *R. subcapitata* probably acclimatized to the presence of Cu and began to grow, although at a low rate (µ = 0.044/h) compared to the control; the t*_d_* in the presence of 33 µg/L Cu was 15.8 h. Algae incubated with 53 µg/L presented a reduced growth rate (µ = 0.012/h), which corresponded to a t*_d_* of 57.8 h, that is, five times higher than the control.

### 3.2. Impact of Cu on Chlorophyll a Content and Photosynthetic Activity

**Figure 2:**
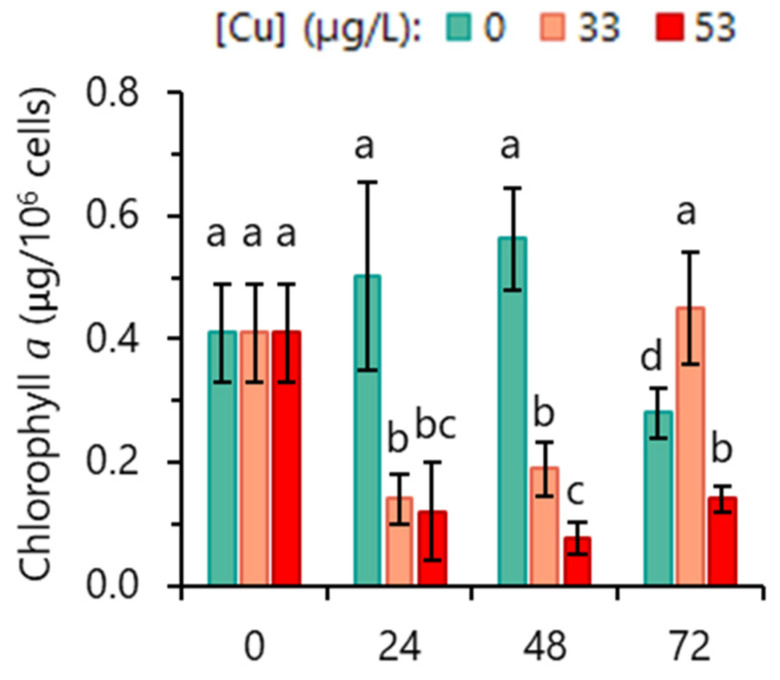
Impact of Cu on *R. subcapitata* chlorophyll *a* content. Data are presented as mean values ± SD; mean values with different letters are significantly different (*p* < 0.05, *n* = 5, Kruskal–Wallis test, Dunn’s post hoc test).

To further characterize the toxic impact of Cu on *R. subcapitata* physiology, its effect on photosynthesis was evaluated. The analysis of chl*a* content revealed a strong reduction (an average of 71%) over time in algae exposed to 53 µg/L Cu (Figure 2). The incubation with 33 µg/L Cu for 24 h induced a decrease of >70% of chl*a* in algal cells. However, as observed with cell growth, subsequent incubation (48–72 h) provoked a recovery of chl*a* content. After incubation with 33 µg/L Cu for 72 h, the algae exhibited a chl*a* content similar to the control at 48 h (Figure 2). In control cultures, chl*a* content reached the maximum values during exponential growth, declining after that (Figure 2).

**Figure 3:**
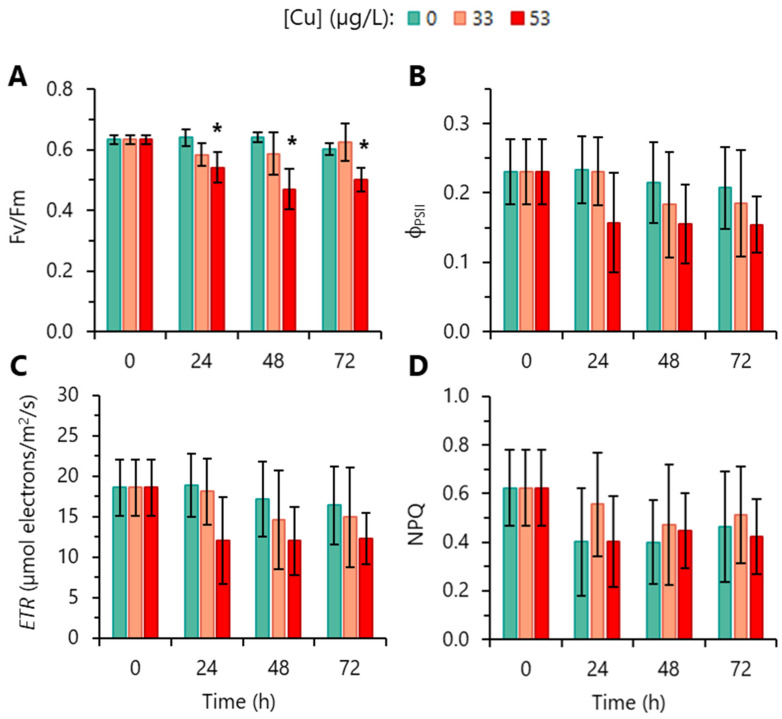
Influence of Cu on *R. subcapitata* photosynthetic activity evaluated by pulse-amplitude-modulation fluorescence assay. (**A**) Maximum photochemical quantum yield of PSII (*F*_v_/*F*_m_). (**B**) Effective photochemical quantum yield of PSII (Φ_PSII_). (**C**) Electron transport rate (*ETR*). (**D**) Non-photochemical quenching (*NPQ*). Data are presented as mean values ± SD. At each time, the statistical difference between control and Cu-treated cells was tested using unpaired Student’s *t*-test; the means with (*) are significantly different from the control (*p* < 0.05, *n* = 5).

The characterization of photosynthetic activity by PAM fluorometry showed that the maximum photochemical quantum yield (*F*_v_/*F*_m_) of PSII was significantly affected in cells exposed to 53 µg/L Cu; the reduction began to be observed at 24 h and continued until 72 h (Figure 3A). The exposure to 33 µg/L Cu did not significantly impact the maximum photosynthetic efficiency (*F*_v_/*F*_m_) of algal cells over the exposure time (72 h) (Figure 3A). Control cultures in the exponential phase of growth displayed a mean value *F*_v_/*F*_m_ of 0.64 (Figure 3A), which is in the range of the values (0.62–0.64) usually reported in *R. subcapitata* [43,44]. Cu, within the concentrations and exposure time evaluated, did not significantly affect other photosynthetic parameters such as the effective quantum yield of PSII (Φ_PSII_) (Figure 3B), photosynthetic electron transport rate (*ETR*) (Figure 3C), and non-photochemical quenching (*NPQ*) (Figure 3D).

### 3.3. ROS Production and Antioxidant Activity

**Figure 4:**
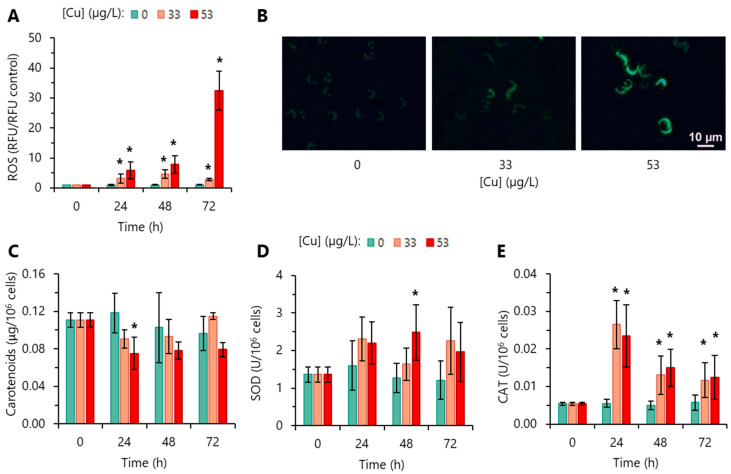
ROS accumulation and antioxidant activity of *R. subcapitata* cells exposed to Cu. (**A**) Reactive oxygen species (ROS) production. (**B**) Visualization of the intracellular accumulation of ROS (green cells) by epifluorescence microscopy and using the H_2_DCFDA probe on algae not exposed or exposed to Cu for 72 h. (**C**) Carotenoids content. (**D**) Superoxide dismutase (SOD) activity. (**E**) Catalase (CAT) activity. Data are presented as mean values ± SD. At each time, the statistical difference between control and Cu-treated cells was tested using unpaired Student’s *t*-test; the means with (*) are significantly different from the control (*p* < 0.05; *n* = 4).

Intracellular accumulation of ROS in algal cells due to the toxic action of Cu was assessed using the redox sensor H_2_DCFDA. After exposure for 24 h to 33 or 53 µg/L Cu, the algae exhibited intracellular ROS levels significantly higher than the control (Figure 4A,B). In the case of incubation with 53 µg/L Cu, ROS levels increased over time and were particularly high (33 times higher than the control) at 72 h (Figure 4A).

Carotenoids (CAR) present a dual function in algal cells: as accessory pigments and as protecting the photosynthetic reaction centres through ROS scavenging [45]. To evaluate the importance of CAR in the fight against ROS, their Cu-induced levels were quantified. Incubation of *R. subcapitata* with 53 µg/L Cu induced a significant reduction in CAR at 24 h (Figure 4C).

To gain further insight into the impact of Cu on antioxidant defenses of *R. subcapitata*, the activity of the two main enzymatic defenses (SOD and CAT) was quantified. The assessment of the activity of these enzymes revealed two different patterns. SOD level was only significantly affected by the exposure to 53 µg/L Cu after 48 h (Figure 4D). CAT levels, for both Cu concentrations, increased significantly over the exposure time (Figure 4E), being particularly high at 24 h. Together, these results evidence a disturbance of the homeostatic mechanisms of the alga.

### 3.4. Lipid Peroxidation and Cell Membrane Integrity

**Figure 5:**
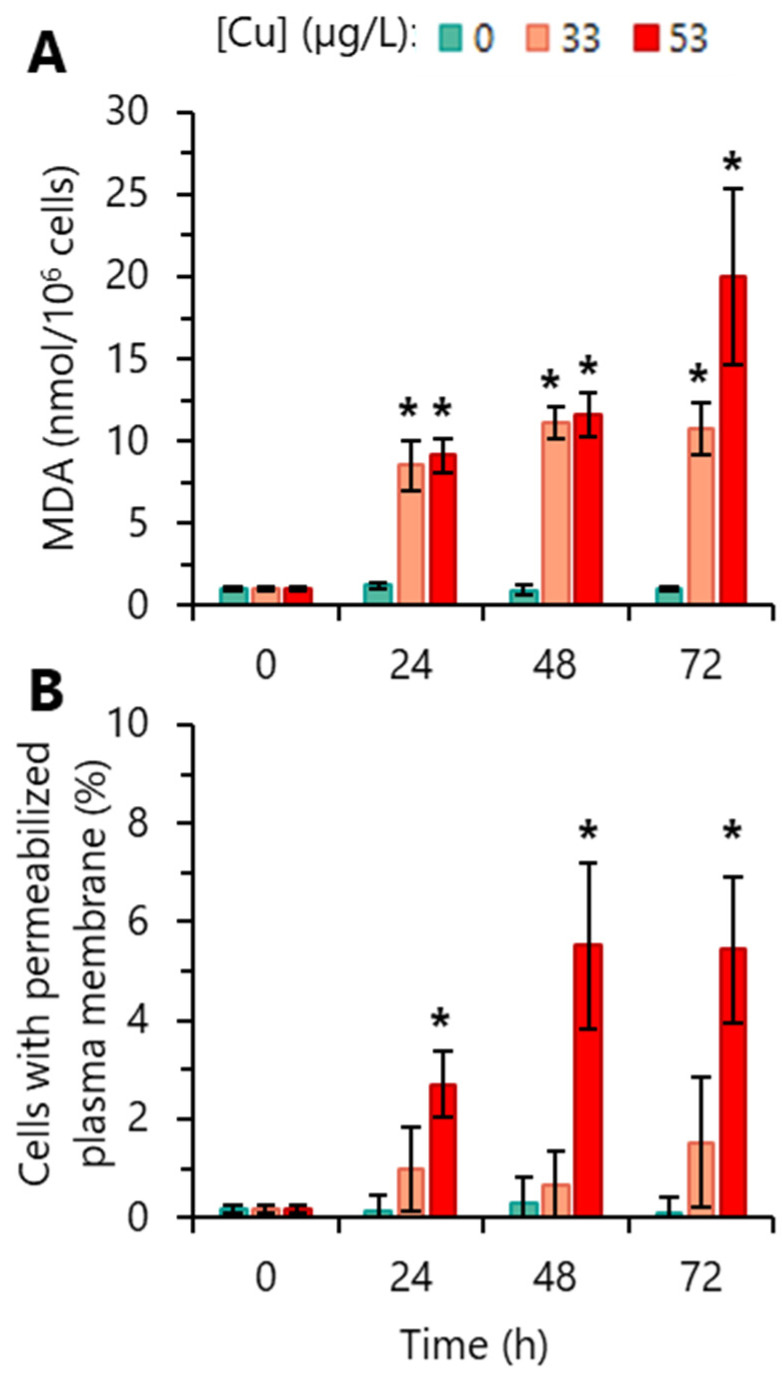
Lipid peroxidation and loss of cell membrane integrity of *R. subcapitata* exposed to Cu. (**A**) Malondialdehyde (MDA) content (lipid peroxidation). (**B**) Loss of plasma membrane integrity. Data are presented as mean values ± SD. At each time, the statistical difference between control and Cu-treated cells was tested using unpaired Student’s *t*-test; the means with (*) are significantly different from the control (*p* < 0.05; *n* = 3 for lipid peroxidation and *n* = 5 for cell membrane integrity).

High levels of intracellular ROS can lead to the oxidative degradation of the lipids (typically polyunsaturated fatty acids, PUFAs) present in cell membranes in a process called lipid peroxidation [46]. To evaluate the level of lipid peroxidation in algal cells induced by exposure to Cu, the levels of MDA (the major end-product of lipid oxidation) were quantified. The incubation of *R. subcapitata* with 33 or 53 µg/L Cu, for 24 h significantly increased MDA levels. Lipid peroxidation increased markedly (20 times higher than basal levels found in the control) in cells incubated with 53 µg/L Cu for 72 h (Figure 5A).

One of the possible impacts of uncontrolled oxidative stress is the injury of cell structures (containing PUFAs), namely plasma membrane, mitochondria, or chloroplasts, due to lipid damage [47]. To better portray the impact of oxidative stress induced by Cu on *R. subcapitata*, the cell membrane integrity of the alga was evaluated. As shown in Figure 5B, the plasma membrane integrity of algae exposed to 33 µg/L Cu was not significantly affected. Conversely, the algal population incubated with 53 µg/L Cu showed a significant increase over time in the number of cells with a compromised plasma membrane (Figure 5B).

## 4. Discussion

Cu is one of the most toxic heavy metals to algae [48]. In the present study, the freshwater microalga *R. subcapitata* was exposed to 33 and 53 µg/L Cu. These Cu concentrations are environmentally relevant since they can be found in surface waters [3]. Even at the low concentrations tested, Cu had a deep impact on algae growth, reducing the number of cells at 72 h by 53 and 93%, respectively. This means that these two copper concentrations correspond to a moderate (33 µg/L) and a high (53 µg/L) toxic impact, assessed through the effect on growth, a universally accepted endpoint to evaluate the toxicity of chemical species [32]. These results prompted us to investigate, in more depth, Cu toxicity on algae. With this aim, Cu’s toxic impacts on sub-cellular processes were evaluated, since these effects remain partially unknown in this freshwater alga.

The evaluation of chlorophyll concentration over time allowed us to observe that cells incubated with 33 µg/L Cu showed a sudden decline of chl*a* content, followed by its recovery. This variation in chlorophyll content fits perfectly into the concept of stress. In response to a stressor, homeostasis is disrupted, initiating an “alarm” signal that leads to a “response” (change in cell metabolism) to restore homeostasis through acclimation [49]. On the contrary, *R. subcapitata* cells exposed to more intense stress (53 µg/L Cu) showed a significant reduction in chl*a* content, without recovery over the 72 h period. The decrease in chl*a* was observed in different algae exposed to Cu: *C. reinhardtii* [50], *P. tricornutum* [51], *R. subcapitata* [52], and *Scenedesmus* sp. [11]. Probably, the reduction in chlorophyll content is a common symptom of heavy metal toxicity in plants and algae as a consequence of the inhibition of their biosynthesis or the induction of their degradation [53].

It is known that chl*a*, the main photosynthetic pigment of algae, has a fundamental importance in the photosynthetic process, namely in the absorption of light (in the antenna complex) and in the transfer of energy from the absorbed photons to the reaction centre where they are used for photochemistry [54]. Hence, the reduction in the chl*a* content may have resulted in the deterioration of photosynthetic complexes, which may explain the (observed) decrease in the *F*_v_/*F*_m_ of PSII, a coefficient generally linked to the maximum photosynthetic capacity of cells; a decrease in this parameter indicates photoinhibition and reduction in the number of functional PSII centres [37]. This relationship was observed in cells exposed to 53 µg/L Cu; in such conditions, a strong reduction in chl*a* content and a significant decrease (comparatively to the control) of *F*_v_/*F*_m_ was observed. However, it is important to underline that Cu, at the range of concentrations used, did not have a significant impact on Φ_PSII_, a parameter that reflects the proportion of light absorbed by the chlorophylls of the PSII antenna that is used photochemically to produce energy [37]. Similarly, in the marine diatom *Nitzschia closterium*, photosynthesis was unaffected at Cu levels up to 153 µg/L [55].

Cu can induce HO· formation via the Haber–Weiss/Fenton reaction in the presence of H_2_O_2_ [56]. Here, we observed that *R. subcapitata* exposed to 33 µg/L or 53 µg/L Cu presented increased intracellular levels of ROS, which elevated hugely over time in the presence of 53 µg/L Cu. In the same way, ROS overproduction has been observed in algae exposed to Cu, usually at higher concentrations. Examples of these microalgae are *C. reinhardtii* [18,19,20], *P. subcapitata* [22], and *P. tricornutum* [16].

ROS can be generated at different sub-cellular levels, namely in peroxisomes, mitochondria, and chloroplasts [57]. In the case of chloroplasts, ROS can be produced in PSII, when the delivery of energy from the excited chlorophylls to the PSII reaction centre exceeds the electron transport capacity between the photosystems due to electron flow limitations at the level of the transport chain; the leaked electrons react with oxygen leading to ROS formation [54]. In *R. subcapitata* exposed to up 53 µg/L Cu, this possibility of ROS formation seems to be ruled out, since the *ETR* values (an indicator of the rate at which electrons are transferred from PSII to PSI) [58] are not significantly different from the control. Thus, although the involvement of chloroplasts in Cu-stressed cells in ROS production cannot be excluded, the photosynthetic electron transport chain (between the two photosystems) does not seem to be the primary source of ROS. Mitochondria can be a plausible candidate. Consistent with this possibility, the modification of mitochondrial membrane potential [13] has been described in *R. subcapitata* exposed to 80 µg/L Cu for 72 h, which was accompanied by an increase in intracellular ROS production [23].

Membrane PUFAs are possible targets of peroxidation by intracellular ROS [59]. Consistent with this possibility, it was observed that algae exposed to 33 or 53 µg/L Cu showed increased levels of ROS and MDA, a biomarker of lipid peroxidation [59]. For the Cu concentrations tested, MDA content seems to be correlated (R^2^ = 0.76–0.84) with ROS overproduction (Figure S1). Likewise, lipid peroxidation has been described in *Scenedesmus* sp. exposed to 159 or 636 µg/L Cu for 6 h or 7 days [24].

On the other hand, lipid peroxidation can result in the loss of membrane integrity [59]. In fact, in cells exposed to 53 µg/L Cu, the loss of plasma membrane integrity appears to be correlated (R^2^ = 0.79) with the level of lipid peroxidation (Figure S2). For this concentration, Cu had a slight algicidal effect (~5%, at 72 h). Overproduction of ROS accompanied by the loss of cell membrane integrity was also observed in *P. tricornutum*, when exposed to ≥2.5 mg/L Cu for 48–96 h [51]. In addition, the algicidal effect (death of algae due to the loss of membrane integrity) in Cu-exposed cells of *Chlamydomonas moewusii* (≥10 mg/L, 24 h) and *C. vulgaris* (≥30 mg/L, 3–24 h) has been described [60]. However, the loss of cell membrane integrity was not observed in algae exposed to a lower concentration of Cu. Cells incubated with 33 µg/L Cu presented intracellular levels of ROS and MDA significantly higher than the control, without loss of cell membrane integrity. Probably, lipids other than those from the plasma membrane, such as those present in chloroplast membranes, rich in PUFAs [57], were potential peroxidation targets. The exposure to oxidative stress, being Cu-induced, lead to an increase in CAT activity to deplete intracellular oxidants and thus, counteract the increase in ROS levels and protect the algal cells. In recent years, the altered expression of genes encoding ROS-scavenging enzymes, such as SOD and CAT, has been described. Thus, the Cu-treated freshwater alga *Closterium ehrenbergii* showed an increased expression of CuZn*SOD* genes [61]. Transcriptional analysis in the alga *D. salina*, exposed to Cd, revealed an altered expression of *SOD* genes [62], while the alga *Auxenochlorella protothecoides*, exposed to ultra-high Cd stress, presented upregulated expression of the Mn*SOD* gene and downregulated the expression of Fe*SOD* and *CAT* genes [63].

**Figure 6:**
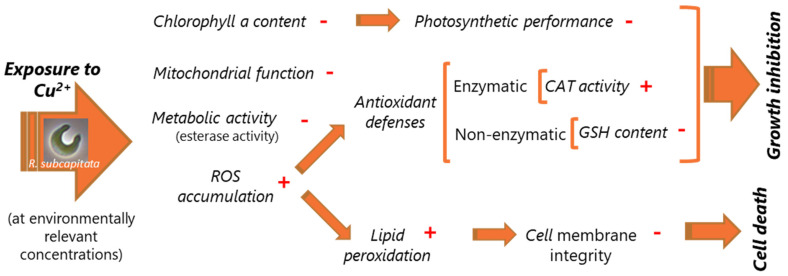
Proposal of the mechanism of action (toxicity pathway) of Cu, at environmentally relevant concentrations on the freshwater alga *R. subcapitata* based on the results here presented and previous works [13,23,27]. Variations in cellular responses are symbolized by “+” or “−“ for an increase or decrease, respectively. CAT—catalase, GSH—reduced glutathione, ROS—reactive oxygen species.

Combining the information here presented with those previously obtained [13,23,27], a proposal of the mechanism of action (toxicity pathway) of Cu on *R. subcapitata* was built, in which the chain of impacts that occur in algal cells is presented (Figure 6). Thus, the combination of several effects such as a decrease in metabolic activity and mitochondrial function [13,23], accompanied by a reduction in chl*a* content and photosynthetic performance and the alteration of enzymatic (CAT) and non-enzymatic defenses, such as reduced glutathione [23] due to the oxidative stress (increase in intracellular ROS accumulation), could explain the inhibition of alga induced by Cu (Figure 6). On the other hand, the increase in the intracellular level of ROS leads to lipid peroxidation and loss of membrane integrity, with the consequent death of the alga cells at higher concentrations of Cu (Figure 6).

## 5. Conclusions

*R. subcapitata* exposed to Cu at environmentally relevant concentrations for up to 72 h presented several toxicity markers depending on metal concentration and exposure time. Thus, algae incubated with 33 µg/L Cu grew more slowly without the loss of cell membrane integrity. A transient decrease in chl*a*, and an increase in ROS and MDA levels were detected in these algae. On the other hand, algae incubated with 53 µg/L Cu showed a greater reduction in growth (93%) and a decrease in chl*a* content and the maximum photochemical quantum yield of PSII. Algae exposed to 53 µg/L Cu also exhibited augmented levels of intracellular ROS, lipid peroxidation, and cells with a compromised plasma membrane.

The elucidation of the mode of action of Cu, at environmentally relevant concentrations, in the alga *R. subcapitata* could be useful in defining strategies to mitigate the toxic effects of Cu, namely through the development of better regulatory guidelines to avoid its release into the environment, thus contributing to the protection of aquatic ecosystems and human health.

## Data Availability

Data will be made available on request.

## Supplementary Materials

The following supporting information can be downloaded at: https://www.mdpi.com/article/10.3390/toxics12120905/s1, Figure S1: Correlation between intracellular ROS accumulation and MDA content in *R. subcapitata*; Figure S2: Correlation between MDA content and % of cells with permeabilized plasma membrane in *R. subcapitata* exposed to 53 µg/L Cu.

## Declarations

Compliance with ethical standards. This article does not contain any studies with human participants or animals performed by the authors.
