# Supplementary material for: Integration of Copper Toxicity Mechanisms in Raphidocelis subcapitata: Advancing Insights at Environmentally Relevant Concentrations
Source: Toxics. 2024 Dec 13;12(12):905. doi: 10.3390/toxics12120905 (PMC11728569; doi:10.3390/toxics12120905)
Supplement: Supplementary file 1 [file toxics-12-00905-s001.zip › toxics-3352927-supplementary.pdf]

Journal Toxics

Supplementary Material

Integration of copper toxicity mechanisms in *Raphidocelis subcapitata*: advancing insights at environmentally relevant concentrations

Manuela D. Machado[1,2,3] and Eduardo V. Soares[1,2,3]*

[1]Bioengineering Laboratory, ISEP, Polytechnic of Porto, Rua Dr António Bernardino de Almeida, 431, 4249-015 Porto, Portugal

[2]CEB - Centre of Biological Engineering, University of Minho, Campus de Gualtar, 4710-057 Braga, Portugal

[3]LABBELS – Associate Laboratory, Braga/Guimarães, Portugal

Manuela D. Machado ORCID: 000-0003-3519-8654

Eduardo V. Soares ORCID: 000-0003-2280-5291

CONTENTS

Figure S1. Correlation between intracellular ROS accumulation and MDA content in *R. subcapitata* S2

Figure S2. Correlation between MDA content and % of cells with permeabilized plasma membrane in *R. subcapitata* exposed to 53 µg/L Cu............................ S2

[*]Corresponding author email address: evs@isep.ipp.pt

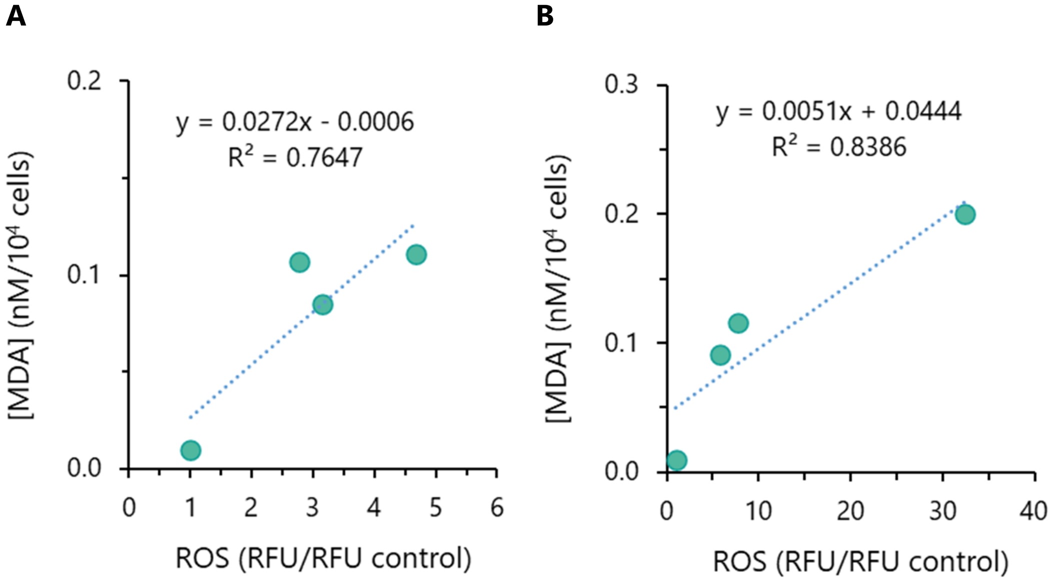

Fig. S1 Correlation between intracellular ROS accumulation and MDA content in *R. subcapitata*. A – cells exposed to 33 µg/L Cu. B – cells exposed to 53 µg/L Cu.

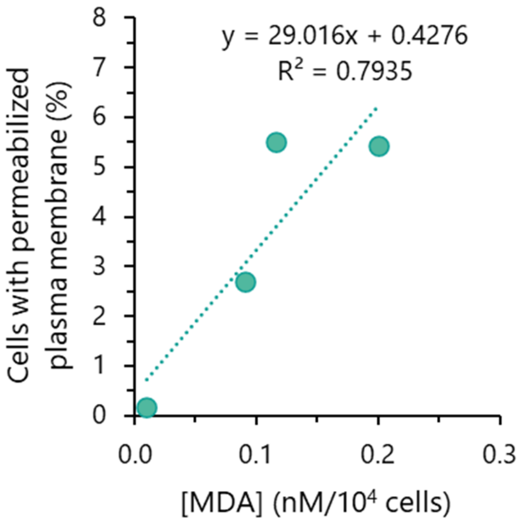

Fig. S2 Correlation between MDA content and percentage of cells with permeabilized plasma membrane in *R. subcapitata* exposed to 53 µg/L Cu.
